# Mental distress in relation to police reporting among adolescent victims of robbery. A population-based study in southern Sweden

**DOI:** 10.1016/j.ssmph.2023.101483

**Published:** 2023-08-06

**Authors:** Maria Fridh, Maria Rosvall, Martin Lindström

**Affiliations:** a Social Medicine and Health Policy, Department of Clinical Sciences in Malmö, Lund University, CRC, Jan Waldenströmsgata 35, Skåne University Hospital Malmö, SE-205 02, Malmö, Sweden; b Center for Primary Health Care Research, Region Skåne and Lund University, S-205 02, Malmö, Sweden; c School of Public Health and Community Medicine, Institute of Medicine, Sahlgrenska Academy, University of Gothenburg, Sweden; d Socialmedicinskt centrum, Regionhälsan, Västra Götalandsregionen, Sweden

## Abstract

• Robberies as well as mental distress have increased among adolescents in Sweden.
• This study on school students in southern Sweden was population-based with a high response rate.
• Half of the robbed adolescents abstained from police reporting.
• Non-reporters had poorer mental health than victims who reported the robbery.
• Non-reporters were also an overall more disadvantaged group.

## Abbreviations

SHC: Subjective Health Complaints
HBSC-SCL: Health Behaviour in School-aged Children symptom checklist

## 1 Introduction

In Sweden, the increase seen in mental distress among young people over the last decades is of great public health concern (Blomqvist et al., 2019). A more recent concern is the increase in street robberies among the young, as shown by self-reported exposure in surveys as well as in police-reported crime (BRÅ, 2021). For example, in Sweden self-reported past-year

robbery victimization increased from 1.5% to 5.9% among boys aged 16–19 years; and for girls, the corresponding increase went from 0.7% to 1.2% between 2016 and 2019 (BRÅ, 2020a). Among 9th grade students (15 years old) exposure to past-year robbery increased from 3% to 4.1% for boys while it remained 1.2% for girls between 2015/17 and 2019 (BRÅ, 2020b). Fear of being robbed increased in the general population during the same time, especially among boys and young men (BRÅ, 2020a). As most robberies take place after school hours in public places where many adolescents gather or pass through (e.g., public transportation hubs, shopping centers, near schools and sports centers) exposure may be hard to avoid (BRÅ, 2021).

Victimization by robbery and similar events can lead to severe short-term and long-term consequences such as mental health problems (e.g., self-blame, loss of trust, interpersonal problems with friends and family, depression, anxiety, PTSD), liberty-restricting avoidance behavior, poorer academic performance (especially if not feeling safe at school), and health-related problems in adulthood (BRÅ, 2021; Finkelhor et al., 2005; Fowler et al., 2009; Gale & Coupe, 2005; Kim et al., 2020; Murray et al., 2018; Phillips, 2021; Posick, 2014; Turanovic & Pratt, 2015). Victims may also react with maladaptive coping behavior such as drug use, delinquency, and offending with an increased risk of revictimization (Fowler et al., 2009; Ruback et al., 2014; Turanovic & Pratt, 2015).

Previous studies have shown associations between robbery victimization and sociodemographic, lifestyle and psychosocial factors (Aho et al., 2016a; BRÅ, 2020b; Engström, 2018; Pusch & Holtfreter, 2021). Associations have also been shown between mental distress and sociodemographic, lifestyle and psychosocial factors (Currie et al., 2012; Finkelhor et al., 2005; Fridh et al., 2018; Ozer et al., 2017; Turanovic & Pratt, 2015).

It is well known that young victims are less likely to report to the police than adult victims (Papp et al., 2019; Watkins, 2005). Psychologically, a victim goes through a three-stage decision-making model of: (a) identifying oneself as a crime victim, (b) determining the seriousness of the crime, and (c) deciding on a course of action (or no action) (Ruback et al., 1984). At each stage the victim is influenced by emotional stress (e.g., related to feelings of injustice and vulnerability to future victimization) and advice from others (e.g., on whether to call or not to call the police) (Ruback et al., 1984). Adolescents may be more reluctant than adults to define a crime episode as serious, criminal, harmful or warranting intervention (Finkelhor et al., 2001). Non-offending adolescents more often regard incidents as crimes than victims who are also offenders (“victim-offenders”) (Zaykowski, 2015). The propensity to report increases if violence is used during the robbery (BRÅ, 2021; Watkins, 2005; Zaykowski, 2015), and there are indications that robberies involving youths in Sweden feature more violence today than 20 years ago (BRÅ, 2021). Parents often mediate between children and the police (Finkelhor et al., 2001), and parental knowledge of victimization has been shown to increase police awareness by nearly five times (Zaykowski, 2015). Older adolescents may decide not to report from concerns about autonomy as well as reliance on peer norms and values that discourage police reporting (Clayman & Skinns, 2012; Finkelhor et al., 2001; Xie & Baumer, 2019). Embarrassment and shame, fear of stigmatization from peers, and feelings of powerlessness may also be barriers to police-reporting (Finkelhor et al., 2001). Fear of retaliation from the offender has been shown to be a significant factor in the decision not to cooperate with the police (Clayman & Skinns, 2012; Papp et al., 2019; Slocum, 2018). Young victims are often threatened with various consequences if they report the crime to the police or refuse to withdraw a police report (BRÅ, 2021), and the victim's perception of threat severity has been shown to predict levels of mental distress (Johansen et al., 2006). In Sweden, one in five adolescents who reported being a victim of robbery to the police later declined to participate in a preliminary investigation (BRÅ, 2021). Explicit threats, usually of violence or death, were seen in two-thirds of the preliminary police investigations of robbery victims younger than 18 years of age in Sweden between 2015 and 2019 (BRÅ, 2021). Regardless of why robbed adolescent abstain from notifying the police, the opportunity of redemption through legal justice is lost. It is important for crime victims to achieve redemption to restore their feeling of personal safety and belief in a just world (Corey et al., 2015; Lench & Chang, 2007; Syropoulos, 2020). Police reporting could therefore reduce some of the mental distress associated with robbery victimization, especially if the perpetrator is prosecuted and legal justice obtained.

A large body of research has investigated the relationship between victimization and police reporting, but as far as we know no study specifically examined the association between robbery victimization and mental distress in relation to police reporting. Our specific focus on robbery victims was motivated by the increase of robberies among young people in Sweden.

The present study aimed to:

1) Explore the characteristics of robbery victims, comparing those who reported the crime to the police with those who did not by sociodemographic, lifestyle and psychosocial factors, exposure to threats, and mental distress.
2) Explore the association between robbery victimization and mental distress in relation to police reporting using non-victims as reference category, with multiple adjustments for relevant covariates.

## 2 Material and methods

### 2.1 Participants and procedures

**Fig. 1:**
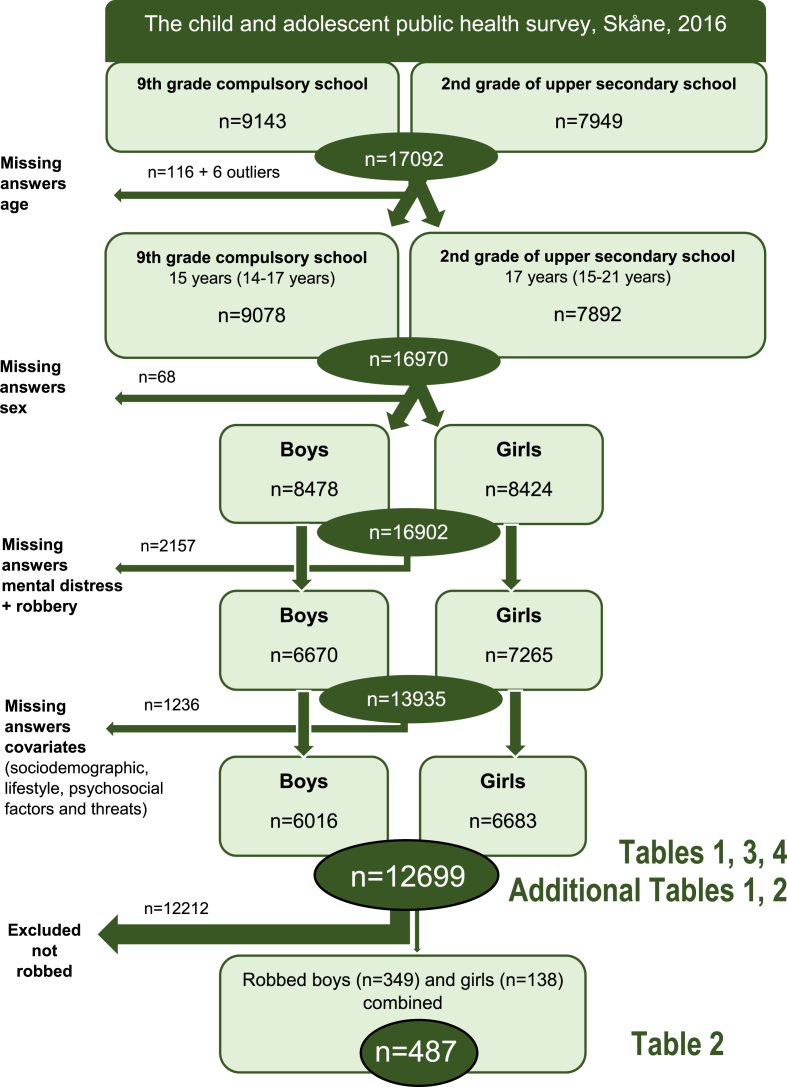
Flowchart of selected study samples.

Skåne is the southernmost part of Sweden and the third largest of 21 regions. In 2016, a total of 1.3 million persons lived in Skåne (13% of the Swedish population), with the age group 15–19 years constituting 5% of the population (the same proportion as in the whole of Sweden) (Statistics Sweden, 2022). This study is based on data derived from a large cross-sectional public health survey conducted by the health authorities in Region Skåne with the aim to map out health, lifestyle and living conditions among school students for public health purposes (Fridh et al., 2016). Students and parents had been informed beforehand that participation was voluntary, confidentiality assured, and that survey results would be used in research. Parental written consent was not necessary as the primary purpose of the survey was not research. The questionnaires were completed anonymously in school (online or by pen and paper) during a one-school-hour timeframe in January 2016 with no follow-up for absentees. More than 27000 students in grade 6 compulsory school (12 years old), grade 9 compulsory school (15 years old) and grade 2 of upper secondary school (17 years old) participated. The majority answered the survey online (80% in 6th grade, 84% in 9th grade and 90% in 2nd grade of upper secondary school). Paper questionnaires were returned to the teacher in an envelope sealed by the student to secure anonymity. According to the Swedish Ethical Review Act (2003) a completed questionnaire is regarded as informed consent for students in grade 9 compulsory school, as children of this age are deemed sufficiently mature to make their own decisions regarding participation in public health surveys. However, as written parental consent is required for research on children younger than 15 years of age students in grade 6 compulsory school were not included in the present study. The 9143 students in 9th grade (response rate 77%) and 7949 in 2nd grade (response rate 73%) comprised a total of 17092 students. Among these, internally missing answers varied from below 1% (sex, age, country of birth), to more than 10% for exposure to different types of violence (bullied 12.4%, seriously threatened 12.7%, robbed 13.0%). Internally missing answers for mental distress (based on eight different subjective health complaints, SHC) was 7.8%. The selected study sample included students in 9th grade compulsory school and 2nd grade of upper secondary school with answers on all covariates used, i.e. 12699 students; 6016 boys (47.4%) and 6683 girls (52.6%) (a flowchart of the study selection is presented in Fig. 1).

### 2.2 Measures

#### 2.2.1 Dependent variable: mental distress measured by subjective health complaints

The general term “subjective health complaints” (SHC) is used to describe a variety of common health symptoms with or without a diagnosis (Haugland & Wold, 2001), and has frequently been employed as a proxy for mental distress among children and adolescents (Fridh, 2018). The public health school survey in Skåne included the widely used, reliable and well-validated instrument Health Behaviour in School-aged Children symptom checklist (HBSC-SCL) (Currie et al., 2012). The students were asked how often they had experienced eight different health complaints (headache, stomachache, backache, feeling low, feeling irritable or bad tempered, feeling nervous, difficulties in getting to sleep, and dizziness) during the last six months, with five answer options for each item from “rarely or never” to “about every day”. Previous studies have employed a variety of different scoring methods for HBSC-SCL, some based on dichotomization of items with varying cut-offs, others on summation of item scores (SHC-index), yet others on summation of item scores followed by dichotomization. In the present study, each health complaint was rated on a five-point frequency scale, ranging from one point for “rarely or never” to five points for “about every day,” generating an index score of 8–40, with higher scores indicating higher levels of mental distress (Carlerby et al., 2011; Fridh et al., 2015). The SHC-index scores were higher for girls (mean 21.4, median 21, mode 22) than boys (mean 16.1, median 15, mode 11), total sample: mean 18.9, median 18, mode 14. The SHC-items showed satisfactory internal consistency with Cronbach's alpha coefficient 0.86 (boys 0.83; girls 0.85). Based on previous research the summarized score was dichotomized, i.e., the highest symptom quartile versus the three lowest quartiles (Carlerby et al., 2011). The cut-off point was set at 24 points, categorizing 13.2% of boys and 38.0% of girls (26.3% total) as mentally distressed.

#### 2.2.2 Independent variable: robbed past year and reported to the police or not

Students were asked if they had been robbed during the past 12 months (yes/no), and if so whether this incident was reported to the police (yes/no). A three-category variable was created: Robbed past 12 months: no = *non-victims*; yes, police reported = *robbed reporters*; yes, not police reported = *robbed non-reporters*. The robbery victims attended schools in all 33 municipalities in Skåne, but most in the two largest, i.e.; Malmö (26.5%) and Helsingborg (17.5%).

#### 2.2.3 Covariates

Based on previous literature (Aho et al., 2016a; BRÅ, 2020b; Currie et al., 2012; Engström, 2018; Finkelhor et al., 2005; Fridh et al., 2018; Ozer et al., 2017; Pusch & Holtfreter, 2021; Turanovic & Pratt, 2015) the following covariates were included in the present study and treated as potential confounders: *Parental occupation* (both parents working/one or neither parent working); *Country of birth* (youth born in Sweden/other country); *Daily smoking* (youth smoking cigarettes every day/less often); *Intense alcohol consumption* (youth drinking alcohol equivalent to at least four cans of strong beer *or* strong cider/alcoholic soft drink, *or* six cans of medium strong beer *or* a whole bottle of wine *or* 25 cl of hard liquor (approximately six shots or drinks) in one session at least once a month past 12 months/less often); *Use of narcotics* (yes if youth had used any of the listed substances “hashish, marijuana, Spice, amphetamines, ecstasy, LSD, cocaine, heroin, GHB, or the like” during the past 12 months/no); *Communication with parents* (easy/not easy to talk to parents when having a problem or just wanting to talk). In addition, students were asked how often they had been *bullied in school* during the past few months (using the regular cut-off ‘‘2 or 3 times a month” (Currie et al., 2012) and whether they had been *seriously threatened by another person* during the past 12 months (yes/no).

### 2.3 Data analysis

The public health survey in Skåne was answered by 17092 students in 9th grade compulsory school and 2nd grade of upper secondary school attending 279 schools; 192 compulsory schools and 88 upper secondary schools (one school included both students in 9th grade compulsory school and 2nd grade of upper secondary school). Due to the hierarchical structure of the data, with the adolescents (first level) nested within schools (second level), multilevel modelling based on within-group and between-group variances for SHC-index was first performed in SPSS. The intra-class correlation (ICC) was calculated as: ICC = school variance/(school variance + individual level variance) *100. The multilevel analyses in SPSS showed only a small clustering effect on school level regarding SHC-index (3.0% ranging from 1.6% in 9th grade compulsory school to 4.2% in 2nd grade of upper secondary school), which indicates that the school context only slightly affects psychological and somatic symptoms. Therefore, all statistical analyses were conducted in ordinary logistic regression models.

The prevalence of robbery victimization was similar among students in 9th grade compulsory school and 2nd grade of upper secondary school for boys and girls, respectively (Table A.1). Answers from the two school grades were therefore combined to increase statistical power. Sex-stratification was motivated by a higher prevalence of mental distress among girls and a higher prevalence of robbery victimization among boys. When comparing robbed students only, answers from boys and girls were combined due to the low number of robbery victims (n = 487).

Table 1 illustrates the differences in background characteristics between the three categories of non-victims, robbed reporters and robbed non-reporters in the total study sample as well as separately for boys and girls. Statistically significant differences were determined by Chi-square tests. Table 2 presents the subsample of robbed students (boys and girls combined) and illustrates the differences in background characteristics between those who reported the crime to the police and not. Statistically significant differences between robbed reporters and non-reporters were determined by Chi-square tests. The bivariate associations between mental distress and covariates were investigated using age-adjusted binary logistic regression (Table 3), and the bivariate associations between robbery victimization and covariates were likewise investigated (Table A.2). The association between robbery victimization and mental distress according to police reporting was investigated by multiadjusted binary logistic regression analyses (Table 4). Model 0 (crude) adjusted for age, Model 1 further adjusted for sociodemographic factors (parental occupation, country of birth), Model 2 further adjusted for lifestyle factors (smoking, alcohol, narcotics), Model 3 further adjusted for psychosocial factors (parental communication, bullying victimization), and Model 4 further adjusted for exposure to serious threat. The statistical analyses were performed using IBM SPSS Statistics version 25.

**Table 1:** **Descriptive statistics of study variables** Students in 9th grade compulsory school (∼15 years) and 2nd grade of upper secondary school (∼17 years) combined. The child and adolescent public health survey, Skåne, 2016.

	Boys				Girls				Total			
	n = 6016 (47.4%)				n = 6683 (52.6%)				n = 12699 (100%)			
	Non-victims	Robbed reporters	Robbed non-reporters	*p-value*^a^	Non-victims	Robbed reporters	Robbed non-reporters	*p-value*^a^	Non-victims	Robbed reporters	Robbed non-reporters	*p-value*^a^
	n = 5667	n = 172	n = 177		n = 6545	n = 54	n = 84		n = 12212	n = 226	n = 261	
	94.2%	2.9%	2.9%		97.9%	0.8%	1.3%		96.2%	1.8%	2.1%	
Both parents work	**77.7%***n = 4401*	**75.6%***n = 130*	**68.4%***n = 121*	0.012*	**76.1%** *n = 4978*	**66.7%***n = 36*	**59.5%***n = 50*	0.001**	**76.8%***n = 9379*	**73.5%** *n = 166*	**65.5%***n = 171*	<0.001***
Born in Sweden	**88.6%** *n = 5023*	**85.5%** *n = 147*	**81.9%***n = 145*	0.011*	**88.1%***n = 5767*	**79.6%***n = 43*	**76.2%***n = 64*	0.001**	**88.4%***n = 10790*	**84.1%** *n = 190*	**80.1%***n = 209*	<0.001***
Daily smoking	**4.0%** *n = 229*	**9.9%***n = 17*	**16.4%***n = 29*	<0.001***	**5.1%***n = 334*	**7.4%***n = 4*	**15.5%***n = 13*	<0.001***	**4.6%***n = 563*	**9.3%***n = 21*	**16.1%***n = 42*	<0.001***
Intense alcohol consumption	**20.8%***n = 1180*	**34.9%***n = 60*	**45.8%***n = 81*	<0.001***	**19.3%** *n = 1263*	**33.3%** *n = 18*	**36.9%***n = 31*	<0.001***	**20.0%***n = 2443*	**34.5%***n = 78*	**42.9%***n = 112*	<0.001***
Narcotics past year	**6.8%***n = 387*	**22.7%***n = 39*	**28.2%***n = 50*	<0.001***	**5.1%***n = 332*	**11.1%** *n = 6*	**17.9%***n = 15*	<0.001***	**5.9%***n = 719*	**19.9%***n = 45*	**24.9%***n = 65*	<0.001***
Not easy to speak to parents	**30.2%***n = 1714*	**33.1%***n = 57*	**44.6%***n = 79*	<0.001***	**36.2%***n = 2372*	**40.7%***n = 22*	**54.8%***n = 46*	0.002**	**33.5%***n = 4086*	**35.0%***n = 79*	**47.9%***n = 125*	<0.001***
Bullied in school past few months	**2.9%***n = 167*	**9.9%***n = 17*	**15.3%***n = 27*	<0.001***	**3.7%***n = 244*	**14.8%***n = 8*	**16.7%***n = 14*	<0.001***	**3.4%***n = 411*	**11.1%***n = 25*	**15.7%***n = 41*	<0.001***
Seriously threatened past year	**10.6%** *n = 598*	**66.9%** *n = 115*	**73.4%** *n = 130*	<0.001***	**8.2%** *n = 536*	**50.0%** *n = 27*	**61.9%***n = 52*	<0.001***	**9.3%** *n = 1134*	**62.8%** *n = 142*	**69.7%***n = 182*	<0.001***
Mental distress b	**12.5%***n = 707*	**16.3%***n = 28*	**33.9%***n = 60*	<0.001***	**37.7%***n = 2469*	**44.4%***n = 24*	**58.3%***n = 49*	<0.001***	**26.0%***n = 3176*	**23.0%***n = 52*	**41.8%***n = 109*	<0.001***

Significance levels: *p < 0.05, **p < 0.01, ***p < 0.001.

a Pearson Chi-Square 2-sided.

b Mental distress measured as SHC-index ≥24 (highest quartile).

**Table 2:** **Descriptive statistics of study variables among robbed students** Students in 9th grade compulsory school (∼15 years) and 2nd grade of upper secondary school (∼17 years) combined. The child and adolescent public health survey, Skåne, 2016.

	Robbed		Reported to the police		
	Total		Yes	No	*p-value*^a^
	*n = 487*	%	*n = 226*	*n = 261*	
			(46.4%)	(53.6%)	
**Both parents working**	*337*	69.2	73.5	65.5	***0.062***
**Born in Sweden**	*399*	81.9	84.1	80.1	*0.288*
**Daily smoking**	*63*	12.9	9.3	16.1	0.030*
**Intense alcohol consumption**	*190*	39.0	34.5	42.9	***0.063***
**Narcotics past year**	*110*	22.6	19.9	24.9	*0.194*
**Not easy to speak to parents**	*204*	41.9	35.0	47.9	0.004**
**Bullied in school past few months**	*66*	13.6	11.1	15.7	*0.146*
**Seriously threatened past year**	*324*	66.5	62.8	69.7	*0.123*
**Mental distress**^b^	*161*	33.1	23.0	41.8	<0.001***

p-value in bold = statistical (or close to statistical) significance.

Significance levels: *p < 0.05, **p < 0.01, ***p < 0.001.

a Chi-Square Fisher's exact test 2-sided.

b Mental distress measured as SHC-index ≥24 (highest quartile).

**Table 3:** **Associations between study variables and mental distress**^a^ Age-adjusted bivariate logistic regression analysis. Students in 9th grade compulsory school (∼15 years) and 2nd grade of upper secondary school (∼17 years) combined, stratified by sex. The child and adolescent public health survey, Skåne, 2016.

	Boys				Girls			
	*n = 6016*				*n = 6683*			
	*n*	%	OR	(95% CI)	*n*	%	OR	(95% CI)
**Parents working**								
Both	*4652*	77.3	1.0		*5064*	75.8	1.0	
One or neither	*1364*	22.7	1.7***	(1.4, 2.0)	*1619*	24.2	1.4***	(1.3, 1.6)
**Country of birth**								
Sweden	*5315*	88.3	1.0		*5874*	87.9	1.0	
Other country	*701*	11.7	1.1	(0.9, 1.4)	*809*	12.1	0.9	(0.8, 1.0)
**Daily smoking**								
No	*5741*	95.4	1.0		*6332*	94.7	1.0	
Yes	*275*	4.6	2.1***	(1.6, 2.8)	*351*	5.3	3.5***	(2.8, 4.5)
**Intense alcohol consumption**								
No	*4695*	78.0	1.0		*5371*	80.4	1.0	
Yes	*1321*	22.0	1.6***	(1.3, 1.9)	*1312*	19.6	1.7***	(1.5, 1.9)
**Use of narcotics past year**								
No	*5540*	92.1	1.0		*6330*	94.7	1.0	
Yes	*476*	7.9	2.8***	(2.2, 3.5)	*353*	5.3	2.4***	(2.0, 3.0)
**Easy to speak to parents**								
Yes	*4166*	69.2	1.0		*4243*	63.5	1.0	
No	*1850*	30.8	2.9***	(2.5, 3.6)	*2440*	36.5	2.7***	(2.4, 3.0)
**Bullied in school past few months**								
No	*5805*	96.5	1.0		*6417*	96.0	1.0	
Yes	*211*	3.5	3.4***	(2.5, 4.6)	*266*	4.0	4.0***	(3.1, 5.2)
**Seriously threatened past year**								
No	*5173*	86.0	1.0		*6068*	90.8	1.0	
Yes	*843*	14.0	3.2***	(2.7, 3.8)	*615*	9.2	3.4***	(2.9, 4.1)
**Robbed past year**								
No	*5667*	94.2	1.0		*6545*	97.9	1.0	
Yes	*349*	5.8	2.3***	(1.8, 3.0)	*138*	2.1	1.9***	(1.3, 2.6)

OR = odds ratio; 95% CI = 95% confidence interval; bold = statistical significance.

Significance levels: *p < 0.05, **p < 0.01, ***p < 0.001.

a Mental distress measured as SHC-index ≥24 (highest quartile).

**Table 4:** **Associations between robbery victimization, police reporting, and mental distress**^a^ Multiadjusted bivariate logistic regression analysis. Students in 9th grade compulsory school (∼15 years) and 2nd grade of upper secondary school (∼17 years) combined. The child and adolescent public health survey, Skåne, 2016.

	Model 0 a		Model 1^b^		Model 2 c		Model 3 d		Model 4 e	
**Boys** n = 6016	OR	(95% CI)	OR	(95% CI)	OR	(95% CI)	OR	(95% CI)	OR	(95% CI)
Non-victims (REF)	1.0		1.0		1.0		1.0		1.0	
Robbed reporters (n = 172)	1.3	(0.9, 2.0)	1.3	(0.9, 2.0)	1.1	(0.7, 1.7)	1.0	(0.7, 1.6)	0.6	(0.4, 1.0)
Robbed non-reporters (n = 177)	3.5***	(2.6, 4.9)	3.4***	(2.5, 4.8)	2.7***	(1.9, 3.8)	2.2***	(1.6, 3.2)	1.4	(1.0, 2.0)
**Girls** n = 6683	OR	(95% CI)	OR	(95% CI)	OR	(95% CI)	OR	(95% CI)	OR	(95% CI)
Non-victims (REF)	1.0		1.0		1.0		1.0		1.0	
Robbed reporters (n = 54)	1.3	(0.8, 2.2)	1.3	(0.7, 2.2)	1.2	(0.7, 2.0)	1.0	(0.6, 1.8)	0.7	(0.4, 1.4)
Robbed non-reporters (n = 84)	2.3***	(1.5, 3.6)	2.3***	(1.5, 3.5)	1.9**	(1.2, 3.0)	1.5	(0.9, 2.4)	1.0	(0.6, 1.7)
**Total n** = 12699	OR	(95% CI)	OR	(95% CI)	OR	(95% CI)	OR	(95% CI)	OR	(95% CI)
Non-victims (REF)	1.0		1.0		1.0		1.0		1.0	
Robbed reporters (n = 226)	1.3	(1.0, 1.8)	1.3	(0.9, 1.8)	1.1	(0.8, 1.6)	1.0	(0.7, 1.4)	0.7	(0.5, 1.0)
Robbed non-reporters (n = 261)	3.1***	(2.4, 4.0)	3.0***	(2.3, 3.9)	2.3***	(1.8, 3.1)	1.9***	(1.4, 2.5)	1.2	(0.9, 1.7)

OR = odds ratio; 95% CI = 95% confidence interval; bold = statistically significant.

Significance levels: *p < 0.05, **p < 0.01, ***p < 0.001.

^a^Mental distress measured as SHC-index ≥24 (highest quartile).

a Model 0: Crude (age-adjusted), total study population also adjusted for sex.

b Model 1: Adjusted for sociodemographic factors (parental occupation, country of birth).

c Model 2: Further adjusted for lifestyle (smoking, alcohol, narcotics).

d Model 3: Further adjusted for psychosocial factors (parental communication, bullied in school).

e Model 4: Further adjusted for exposure to serious threats past year.

## 3 Results and discussion

Past-year robbery victimization was experienced by 349 boys (5.8%) and 138 girls (2.1%), and approximately half of these reported the offense to the police (50/50 among boys, fewer ≈ 40/60 among girls).

### 3.1 Characteristics of robbery victims

A general pattern of increasing disadvantage regarding sociodemographics, lifestyle, and psychosocial factors, as well as exposure to serious threats, was seen across the three categories of non-victims, robbed reporters and robbed non-reporters (Table 1).

Table 2 shows the characteristics of robbery victims (n = 487) stratified by police reporting (yes/no), highlighting the statistically significant differences between reporters and non-reporters. Non-reporters were almost twice as often mentally distressed, had significantly more difficulties communicating with parents and were more often daily smokers than police reporting victims. The covariates intense alcohol consumption and not having two working parents were close to statistically more prevalent among non-reporters. Two-thirds of the robbed students had experienced serious threats, but the difference between non-reporters (69.7%) and reporters (62.8%) was not statistically significant.

The results presented in Table 1, Table 2 relate to the first study aim of exploring the characteristics of robbery victims. The general pattern of increasing disadvantage among non-victims, reporters and non-reporters was similar to previous studies on non-victims, victims and offenders (Jennings et al., 2012; Kühlhorn, 1990).

Negative health behaviors tend to co-occur in adolescents, and daily smoking is highly correlated with other risky health behaviors such as alcohol use (Coleman et al., 2014; Zweig et al., 2001). A delinquent lifestyle involving risky health behaviors has been shown to increase the risk of victimization for several crimes including robbery (Jennings et al., 2012; Lauritsen et al., 1991). Those involved in crime as both victims and offenders (“victim-offenders”) have been shown to have the largest number of health-related risk factors compared with non-victims, victims only and offenders only (Reingle & Maldonado-Molina, 2012). In the present study, the proportion of victim-offenders might be higher among robbed non-reporters than reporters. This explanation is merely speculative, however, as no question on offending was included in the survey.

In line with previous research, robbery victims with poor parental communication were less likely to notify the police compared with robbery victims with easy communication (Zaykowski, 2015). Poor parental communication is associated with mental distress among adolescents. Poor communication furthermore reduces the opportunity of parental help in coping with feelings of distress after victimization (Ozer et al., 2017). It is well known that serious parental dysfunction (e.g., mentally ill, substance abusing, or criminal parents) are strong predictors of mental distress in children and adolescents (Anda et al., 2006). When parents are absent the children more often develop harmful lifestyle habits such as binge drinking and substance abuse (Saladino et al., 2021). Alcohol inebriation is known to increase the risk of both victimization and offending (Engström, 2018; Reingle & Maldonado-Molina, 2012). Drug abuse is associated with crimes such as robberies and drug dealing, and there is a strong connection between drug abuse, criminality, and low socioeconomic status among youths (Saladino et al., 2021). A Swedish national survey on 9th grade students showed that 13% of boys who had committed a narcotics offense had also been robbed (i.e., “victim-offenders”) compared with 4% of all boys (BRÅ, 2020b). In disadvantaged neighborhoods where crime is prevalent, victims may be reluctant to contact the police because of fear of retaliation or concerns about bringing unwanted attention to themselves (Berg et al., 2013; Clayman & Skinns, 2012).

### 3.2 Bivariate associations with covariates

The bivariate age-adjusted associations between mental distress and covariates (Table 3) and between robbery victimization and covariates (Table A.2) were all significant, apart from the association between mental distress and country of birth. Robbery victims had approximately doubled odds ratios (ORs) of mental distress compared with non-robbed peers.

### 3.3 Multiadjusted associations between robbery victimization, police reporting, and mental distress

Table 4 presents results relating to the second study aim, i.e., the multiadjusted association between robbery victimization and mental distress according to police reporting. When students were divided into three categories according to experience of robbery and police reporting using non-victims as reference category, only robbed non-reporters showed significantly higher odds ratios of mental distress (age-adjusted Model 0: boys OR 3.5 (95% CI: 2.6, 4.9); girls OR 2.3 (95% CI: 1.5, 3.6)). For boys, the strength of the association decreased but remained significant through Model 1 (sociodemographic factors), Model 2 (lifestyle factors), and Model 3 (psychosocial factors), but statistical significance disappeared in the final model after further adjustment for exposure to past-year serious threats. The largest impact on the association was seen for lifestyle factors and threats. Among girls, the association with mental distress was weaker (lower effect measure (OR) and less statistical power) and was no longer significant after adjustment for psychosocial factors in Model 3.

Lower levels of mental distress among robbed reporters than non-reporters concur with the results of a previous study that evaluated the role of help-seeking on the victimization-psychological distress link in Latino women (Cuevas et al., 2014). In this study, formal help-seeking (reporting to the police) but not informal help-seeking (talking to someone in the family or a friend) was associated with lower psychological distress in lifetime victims of interpersonal violence.

### 3.4 Adolescents’ police-cooperation

Police notification is one of several coping options available to victims (other options are for example to rely on family or friends) (Cuevas et al., 2014; Xie & Baumer, 2019). The decision to report a crime to the police can be described as a cost-benefit calculation based on the perceived benefits of reporting (e.g., protection, property recovery, victim compensation, and perpetrator brought to justice) and the anticipated costs of doing so (e.g., time and effort, feelings of shame or embarrassment, social stigma, or fear of retaliation) (Felson et al., 2002). In the present study, exposure to serious threats might indicate fear of retaliation but the survey did not clarify if the threats were related to the robbery.

Children and adolescent victims are under-researched in the help-seeking literature (Xie & Baumer, 2019). Most research examining youth cooperation with the police has been conducted in the U.S. (Clayman & Skinns, 2012), a country where strained relations between especially poor people of color and the police is a cause of great concern (Slocum, 2018). Trust in the police is somewhat higher in the general population in Sweden (78%) than in the U.S. (73%) (Choi & Kruis, 2021), and strained relations with the police are not generally viewed as an issue of concern or a potential obstacle for police reporting in Sweden. A report can be made in several ways, such as reaching out to a police officer nearby, visiting a police station or contacting the police by phone or online. Children can report to the police without parental help or consent, and detailed information on police reporting and procedure is easily accessible to young persons, e.g., on digital platforms such as Youth Guidance Centre and Victim Support Sweden. If a child has reported a crime on its own, the parents/guardians are usually contacted by the police in the proceedings of investigation.

If the victim perceives the police as unable to intervene effectively, reporting is less likely (Finkelhor et al., 2001). In the 2020 Swedish Crime Survey, 24% of the general popoulation 16–84 years old had been subjected to a crime that was reported to the police in the last three years. Of these, the majority (54%) was very or quite satisfied with the way the police treated them, but only 19% were satisfied with police effectiveness in investigating the crime (BRÅ, 2020a). Among 9th grade students, the proportion agreeing completely/to some extent with the statement that “the police do a good job overall” has fallen slightly among boys (by 5 percentage points to 43%) and girls (by 3 percentage points to 50%) between 2015 and 2019, however about one in four (25% boys and 28% girls) neither agreed nor disagreed with the statement (BRÅ, 2020b).

The increase in robberies in Sweden is a cause of serious concern, and a greater effort is needed to keep young people safe in public places and in school (Aho et al., 2016a; Kim et al., 2020). Fear of crime affects a much larger population than direct victims (Gale & Coupe, 2005; Jackson & Gouseti, 2016), and decreasing all forms of victimization including robbery is a priority that could improve mental health among adolescents in general (Aho et al., 2016a; Murray et al., 2018).

### 3.5 Implications for future research

Future studies would benefit from investigating the association between robbery victimization and mental health in relation to police reporting by even larger population-based study samples to ensure adequate statistical power for sex stratified sub-analyses. For example, a national study in the U.S. showed that economic disadvantage decreased the probability that men reported being robbed to the police but increased the probability of women doing so (Zaykowski et al., 2019).

More detailed questions on the robbery would be valuable (such as location, levels of violence, threats, use of weapon, physical injury, frequency, victim-offender relationship, and help-seeking behavior) (Watkins, 2005; Xie & Baumer, 2019). Different types of violence often co-occur and overlap, and polyvictimization (multiple victimization exposures of different kinds) is a substantial risk factor for both internal (e.g., anxiety, depression, PTSD) and external (e.g., conduct problems, hyperactivity, aggression) mental health problems (Haahr-Pedersen et al., 2020). Future research would furthermore benefit from considering the whole range of different types of violence, including both conventional crime and different types of abuse, to avoid an overestimation of the impact a single type of victimization has on psychopathology outcomes (Aho et al., 2016b; Haahr-Pedersen et al., 2020). Information on the victim's substance abuse, offending, and parental/peer/school factors should also be included for a broad perspective on factors affecting mental health in relation to robberies and police reporting. Causal associations may be determined by using a longitudinal design. Qualitative research would contribute greatly to provide further in-depth understanding on why robbed adolescents refrain from reporting the offense to the police. Qualitative research could also clarify what type of support is most helpful for young robbery victims, as well as for parents who often feel at a loss on how to best support their victimized child (BRÅ, 2021).

### 3.6 Strengths and limitations

The present study is based on a large population-based sample with anonymous self-reports and a high response rate, revealing a better estimate of the true number of robbery victims than official records (BRÅ, 2020b) and providing information on a wide variety of potential confounders. However, several limitations need to be addressed. First, causal conclusions cannot be inferred by a cross-sectional study design. Not reporting a robbery to the police (e.g., because of threats of retaliation) might increase mental distress, but mental distress might also decrease the likelihood of police reporting (e.g., from lack of energy). Second, the true degree of over- or underreporting is unknown, but previous research indicate that answers are more truthful in surveys than face-to-face or telephone interviews, and anonymity reduces the risk of social desirability bias (Klein et al., 2020; Preisendörfer & Wolter, 2014). Third, although adjustments were made for relevant covariates, we lacked information on several important factors as discussed above in section 3.5. Fourth, complete case analysis resulted in exclusion of 25.7% of the eligible sample. Further analysis comparing the excluded students (n = 4393) with included study members (n = 12699) showed that excluded individuals were significantly more often boys, 9th grade students (i.e., younger), sociodemographically disadvantaged, daily smokers, narcotic users, bullied in school, exposed to threats, and robbed (5.9% vs. 3.8%). There were no significant differences between included and excluded study members regarding mental distress, intense alcohol consumption or parental communication. The proportion of robbery victims in the total population of 17092 students (4.1%, boys 6.2% and girls 2.2%) was similar to the selected study sample (3.8%, boys 5.8% and girls 2.1%), and the same proportion of robbery victims reported the crime to the police (46%). A complete case analysis was preferred since multiple imputation presume missing at random for the included covariates (Little et al., 2022; Sterne et al., 2009), and we do not know if all the variables in our study were missing at random. The substantial loss of the original sample causes a substantial loss of precision and power. The results of this study need to be interpreted in consideration of these circumstances.

### 3.7 Conclusions

This study showed that approximately half of the adolescent robbery victims in southern Sweden had not reported the offense to the police. Compared with police reporting victims they had poorer mental health and were overall a more disadvantaged group. Further research is needed to elucidate reasons for non-reporting and underlying associations with mental distress.

## Ethical statement

The Regional Ethical Committee in Lund, Sweden approved this study (Dnr 2016/431).

## Author contribution

Maria Fridh: Conceptualization, Formal analysis, Writing – Original draft, Writing – Reviewing and editing. Maria Rosvall: Reviewing and editing. Martin Lindström: Conceptualization, Reviewing and editing, Funding acquisition.

## Funding

This work was supported by the Swedish ALF Government 2023–2026 and the 10.13039/501100004359Swedish Research Council (Vetenskapsrådet) [Dnr 2019-01631].

## Declaration of competing interest

The authors do not have any potential conflicts of interest or financial disclosures to report.

## Data Availability

The authors do not have permission to share data.
