# Supplementary material for: Mental distress in relation to police reporting among adolescent victims of robbery. A population-based study in southern Sweden
Source: SSM Popul Health. 2023 Aug 6;23:101483. doi: 10.1016/j.ssmph.2023.101483 (PMC10425401; doi:10.1016/j.ssmph.2023.101483)
Supplement: Multimedia component 1 [file mmc1.docx]

| **Table A.1. Descriptive statistics of study variables stratified by school year**  Boys and girls in 9th grade compulsory school (~15 years) and 2nd grade of upper secondary school (~17 years), respectively.  The child and adolescent public health survey, Skåne, 2016. | | | | | | | | | | | | | | | | | | |
| --- | --- | --- | --- | --- | --- | --- | --- | --- | --- | --- | --- | --- | --- | --- | --- | --- | --- | --- |
|  |  | **Boys** | | | | | | | |  | **Girls** | | | | | | | |
|  |  | *n=6016* | | | | | | | |  | *n=6683* | | | | | | | |
|  |  | Total | |  | *School year* | | |  | *p-value ᵃ* |  | Total | |  | *School year* | | |  | *p-value ᵃ* |
|  |  | *n* | % |  | 9 |  | 11 |  |  |  | *n* | % |  | 9 |  | 11 |  |  |
|  |  |  |  |  | *n=3217* |  | *n=2799* |  |  |  |  |  |  | *n=3493* |  | *n=3190* |  |  |
|  |  |  |  |  | 53.5% |  | 46.5% |  |  |  |  |  |  | 52.3% |  | 47.7% |  |  |
| **Parents working** | | | | | | | | | | | | | | | | | | |
|  | Both | *4652* | 77.3 |  | **76.9** |  | **77.8** |  |  |  | *5064* | 75.8 |  | **75.4** |  | **76.2** |  |  |
|  | One or neither | *1364* | 22.7 |  | **23.1** |  | **22.2** |  | *0.405* |  | *1619* | 24.2 |  | **24.6** |  | **23.8** |  | *0.440* |
| **Country of birth** | | | | | | | | | | | | | | | | | | |
|  | Sweden | *5315* | 88.3 |  | **87.5** |  | **89.3** |  |  |  | *5874* | 87.9 |  | **88.1** |  | **87.6** |  |  |
|  | Other country | *701* | 11.7 |  | **12.5** |  | **10.7** |  | ***0.036**** |  | *809* | 12.1 |  | **11.9** |  | **12.4** |  | *0.523* |
| **Daily smoking** | | | | | | | | | | | | | | | | | | |
|  |  | *275* | 4.6 |  | **2.9** |  | **6.5** |  | ***<0.001****** |  | *351* | 5.3 |  | **3.7** |  | **6.9** |  | ***<0.001****** |
| **Intense alcohol consumption** | | | | | | | | | | | | | | | | | | |
|  |  | *1321* | 22.0 |  | **10.3** |  | **35.3** |  | ***<0.001****** |  | *1312* | 19.6 |  | **10.0** |  | **30.1** |  | ***<0.001****** |
| **Narcotics past year** | | | | | | | | | | | | | | | | | | |
|  |  | *476* | 7.9 |  | **4.7** |  | **11.6** |  | ***<0.001****** |  | *353* | 5.3 |  | **3.0** |  | **7.7** |  | ***<0.001****** |
| **Not easy to speak to parents** | | | | | | | | | | | | | | | | | | |
|  |  | *1850* | 30.8 |  | **29.0** |  | **32.7** |  | ***0.002***** |  | *2440* | 36.5 |  | **36.7** |  | **36.3** |  | *0.780* |
| **Bullied in school past few months** | | | | | | | | | | | | | | | | | | |
|  |  | *211* | 3.5 |  | **4.2** |  | **2.7** |  | ***0.002***** |  | *266* | 4.0 |  | **5.6** |  | **2.2** |  | ***<0.001****** |
| **Seriously threatened past year** | | | | | | | | | | | | | | | | | | |
|  |  | *843* | 14.0 |  | **13.5** |  | **14.6** |  | *0.193* |  | *615* | 9.2 |  | **9.8** |  | **8.6** |  | *0.083* |
| **Robbed past year** | | | | | | | | | | | | | | | | | | |
|  | Yes | *349* | 5.8 |  | **5.7** |  | **5.9** |  | *0.782* |  | *138* | 2.1 |  | **2.1** |  | **2.0** |  | *0.931* |
| **Robbed past year** | | | | | | | | | | | | | | | | | | |
|  | No | *5667* | 94.2 |  | **94.3** |  | **94.1** |  |  |  | *6545* | 97.9 |  | **97.9** |  | **98.0** |  |  |
|  | Yes, reported | *172* | 2.9 |  | **2.9** |  | **2.9** |  |  |  | *54* | 0.8 |  | **0.7** |  | **0.9** |  |  |
|  | Yes, not reported | *177* | 2.9 |  | **2.9** |  | **3.0** |  | *0.921* |  | *84* | 1.3 |  | **1.4** |  | **1.1** |  | *0.456* |
| **Mental distress ᵇ** | | | | | | | | | | | | | | | | | | |
|  |  | *795* | 13.2 |  | **12.0** |  | **14.6** |  | ***0.002***** |  | *2542* | 38.0 |  | **36.1** |  | **40.2** |  | ***0.001***** |
|  |  |  |  |  |  |  |  |  |  |  |  |  |  |  |  |  |  |  |
| p-value in bold = statistical significance.  Significance levels: * p < 0.05, ** p < 0.01, *** p < 0.001.  ᵃ Pearson Chi-Square 2-sided for variables with more than two categories, and Chi-Square Fisher’s exact test 2-sided for binary variables.  ᵇ Mental distress measured as SHC-index ≥ 24 (highest quartile). | | | | | | | | | | | | | | | | | | |
