# Supplementary material for: Mental distress in relation to police reporting among adolescent victims of robbery. A population-based study in southern Sweden
Source: SSM Popul Health. 2023 Aug 6;23:101483. doi: 10.1016/j.ssmph.2023.101483 (PMC10425401; doi:10.1016/j.ssmph.2023.101483)
Supplement: Multimedia component 2 [file mmc2.docx]

| **Table A.2. Associations between study variables and robbery victimization**  Age-adjusted bivariate logistic regression analysis.  Students in 9th grade compulsory school (~15 years) and 2nd grade of upper secondary school (~17 years) combined, stratified by sex.  The child and adolescent public health survey, Skåne, 2016. | | | | | | | | | | | | |
| --- | --- | --- | --- | --- | --- | --- | --- | --- | --- | --- | --- | --- |
|  |  | **Boys** | | | | |  | **Girls** | | | | |
|  |  | *n=6016* | | | | |  | *n=6683* | | | | |
|  |  | *n* | % |  | OR | (95 % CI) |  | *n* | % |  | OR | (95 % CI) |
| **Parents working** | | | | | | | | | | | | |
|  | Both | *4652* | 77.3 |  | 1.0 |  |  | *5064* | 75.8 |  | 1.0 |  |
|  | One or neither | *1364* | 22.7 |  | **1.3*** | (1.1, 1.7) |  | *1619* | 24.2 |  | **1.9***** | (1.4, 2.7) |
| **Country of birth** | | | | | | | | | | | | |
|  | Sweden | *5315* | 88.3 |  | 1.0 |  |  | *5874* | 87.9 |  | 1.0 |  |
|  | Other country | *701* | 11.7 |  | **1.5**** | (1.1, 2.0) |  | *809* | 12.1 |  | **2.2***** | (1.4, 3.2) |
| **Daily smoking** | | | | | | | | | | | | |
|  | No | *5741* | 95.4 |  | 1.0 |  |  | *6332* | 94.7 |  | 1.0 |  |
|  | Yes | *275* | 4.6 |  | **3.5***** | (2.5, 4.9) |  | *351* | 5.3 |  | **2.6***** | (1.6, 4.5) |
| **Intense alcohol consumption** | | | | | | | | | | | | |
|  | No | *4695* | 78.0 |  | 1.0 |  |  | *5371* | 80.4 |  | 1.0 |  |
|  | Yes | *1321* | 22.0 |  | **2.6***** | (2.1, 3.3) |  | *1312* | 19.6 |  | **2.5***** | (1.7, 3.6) |
| **Use of narcotics past year** | | | | | | | | | | | | |
|  | No | *5540* | 92.1 |  | 1.0 |  |  | *6330* | 94.7 |  | 1.0 |  |
|  | Yes | *476* | 7.9 |  | **4.6***** | (3.5, 6.0) |  | *353* | 5.3 |  | **3.4***** | (2.1, 5.6) |
| **Easy to speak to parents** | | | | | | | | | | | | |
|  | Yes | *4166* | 69.2 |  | 1.0 |  |  | *4243* | 63.5 |  | 1.0 |  |
|  | No | *1850* | 30.8 |  | **1.5**** | (1.2, 1.8) |  | *2440* | 36.5 |  | **1.7**** | (1.2, 2.4) |
| **Bullied in school past few months** | | | | | | | | | | | | |
|  | No | *5805* | 96.5 |  | 1.0 |  |  | *6417* | 96.0 |  | 1.0 |  |
|  | Yes | *211* | 3.5 |  | **4.8***** | (3.4, 6.8) |  | *266* | 4.0 |  | **5.0***** | (3.1, 8.0) |
| **Seriously threatened past year** | | | | | | | | | | | | |
|  | No | *5173* | 86.0 |  | 1.0 |  |  | *6068* | 90.8 |  | 1.0 |  |
|  | Yes | *843* | 14.0 |  | **19.9***** | (15.6, 25.4) |  | *615* | 9.2 |  | **15.0***** | (10.6, 21.3) |
| **Mental distress ^a^** | | | | | | | | | | | | |
|  | No | *5221* | 86.8 |  | 1.0 |  |  | *4141* | 62.0 |  | 1.0 |  |
|  | Yes | *795* | 13.2 |  | **2.3***** | (1.8, 3.0) |  | *2542* | 38.0 |  | **1.9***** | (1.3, 2.6) |
| OR = odds ratio; 95% CI = 95% confidence interval; bold = statistical significance.  Significance levels: * p < 0.05, ** p < 0.01, *** p < 0.001.  **^a^** Mental distress measured as SHC-index ≥ 24 (highest quartile). | | | | | | | | | | | | |
